# “Dad is feeling blue”: what to know about paternal perinatal depression

**DOI:** 10.1192/j.eurpsy.2022.1224

**Published:** 2022-09-01

**Authors:** C. Peixoto, D. Rego, M. Cruz, H. Medeiros

**Affiliations:** Hospital do Divino Espírito Santo de Ponta Delgada, Psychiatry, Ponta Delgada, Portugal

**Keywords:** postpartum depression, Paternal perinatal depression

## Abstract

**Introduction:**

The transition into parenthood is associated with an increased psychopathological vulnerability. Most studies have focused on mothers, although recently, the role of fathers has grown more importance. Paternal perinatal depression (PPD) is an episode of major depressive disorder occurring in new or expectant fathers during the perinatal period. PPD is not widely acknowledged and research are rare.

**Objectives:**

The authors intend to review the literature about PPD, focusing on its prevalence, risk factors, clinical features, treatment and consequences.

**Methods:**

Non-systematic review of the literature through PubMed.

**Results:**

A meta-analysis of PPD estimated a prevalence of 10.4%. Risk factors of PPD are multiple and complex. There are sociodemographic factors, such as marital status, monthly income and social support. Psychological factors, for instance history of depression, maternal prenatal anxiety and maternal depression. Some literature also suggests hormonal changes on men like increase estrogen and lower testosterone levels. PPD can present with symptoms of mood alterations, like irritability and restricted emotions, anxiety, fatigue, insomnia, loss of appetite. Also common are behavioural disturbances such as interpersonal conflicts, impulsivity, violence, avoidance behaviour, and substance abuse. There are no studies to specific treatments to PPD, so the treatment is the same for women, such as antidepressants and psychotherapy. If untreated, PPD can have an adverse influence on the health and wellbeing of the mother and child.

**Conclusions:**

PPD is still underscreened, underdiagnosed and undertreated. It is fundamental identifying risk factors and the development of specific interventions. Further research on PPD is needed.

**Disclosure:**

No significant relationships.

